# Mesenchymal stromal/stem cell-derived exosomes and genitourinary cancers: A mini review

**DOI:** 10.3389/fcell.2022.1115786

**Published:** 2023-01-04

**Authors:** Aria Salehpour, Saule Balmagambetova, Nadiar Mussin, Asset Kaliyev, Farhad Rahmanifar

**Affiliations:** ^1^ PerciaVista R&D Co.Ltd, Shiraz, Iran; ^2^ Department of Oncology, West Kazakhstan Marat Ospanov Medical University, Aktobe, Kazakhstan; ^3^ Department of Surgery No. 2, West Kazakhstan Medical University, Aktobe, Kazakhstan; ^4^ Department of Basic Sciences, School of Veterinary Medicine, Shiraz University, Shiraz, Iran

**Keywords:** Mesenchymal Stromal/Stem Cell, MSCs, Exosomes, miRNAs, Urogenital neoplasms, Genitourinary neoplasms, Reproductive neoplasms, Reproductive cancers

## Abstract

Mesenchymal stromal/stem cell- (MSC-) derived exosomes are gaining popularity for their involvement in tissue repair and repressing various tumors through extensive patterns. Nevertheless, the impact of extracellular vesicles produced by stem cells on tumor formation and progression is controversial and seems to depend on several factors. The utilization of MSCs’ various capabilities in urogenital neoplasms is widely regarded as a potential future therapeutic as well. These genitourinary neoplasms include prostatic neoplasms, ovarian neoplasms, cervical neoplasms, endometrial neoplasms, bladder neoplasms, and renal cell neoplasms. The present study has concentrated on the most recent information on genitourinary neoplasms employing MSCs derived exosomes’ many capabilities, such as delivering effective RNAs, extensive tissue compatibility, and specificity with tumor identification without inherent limitations of cell therapy.

## Mesenchymal stromal/stem cell (MSC-) derived exosomes and genitourinary neoplasms

MSCs are a population of multipotent cell lineage that may differentiate into distinct cell types and are found in several organs (Pittenger et al., 1999; Mehrabani et al., 2015). In addition to having the ability to divide themselves repeatedly, they also have the ability to engage directly with numerous immune cells to exhibit anti-inflammatory and immunosuppressive effects (Wang et al., 2014; Tamadon et al., 2015).

Previously exosomes have been widely studied and proposed as a tool for diagnosing various genitourinary neoplasms including ovarian cancer (Taylor and Gercel-Taylor, 2008), endometrial cancer (Maida et al., 2016), cervical cancer (Honegger et al., 2015), and prostate cancer (Vlaeminck-Guillem, 2018). Exosomes, membrane-bound vesicles with a diameter of a few nanometers, are certainly one of the most biocompatible forms of inter-cell messengers for regenerative medicine and medication administration (Hood, 2016).

Exosomes produced from MSCs (MSC-EXOs) are effective therapeutic carriers, and they seem to replicate the wide therapeutic benefits seen in MSCs (Madrigal et al., 2014; Khajehahmadi et al., 2016; Bazoobandi et al., 2020; Xunian and Kalluri, 2020). Previous data have suggested that MSCs may settle within tumors shown by the detection of MSCs integrated into tumors after injection (Kidd et al., 2009).

MSC-EXOs are of growing interest due to their role in tissue healing, which includes reducing inflammation and boosting the recovery of damaged tissue. However, the effect of stem cell-derived extracellular vesicles (EVs) on tumor development and progression is debatable and seems to be influenced by the EV source, the kind of tumor, and the mode of exosome therapy (Bruno et al., 2017; Vakhshiteh et al., 2019). When compared to liposomes of equivalent size, integration of exosomes into tumor cells is 10 times higher, demonstrating the improved selectivity of exosomes for tumor identification (Smyth et al., 2014), and compared to regular cells, tumor cells are more capable of internalizing exosomes (Greco et al., 2016).

**FIGURE 1:**
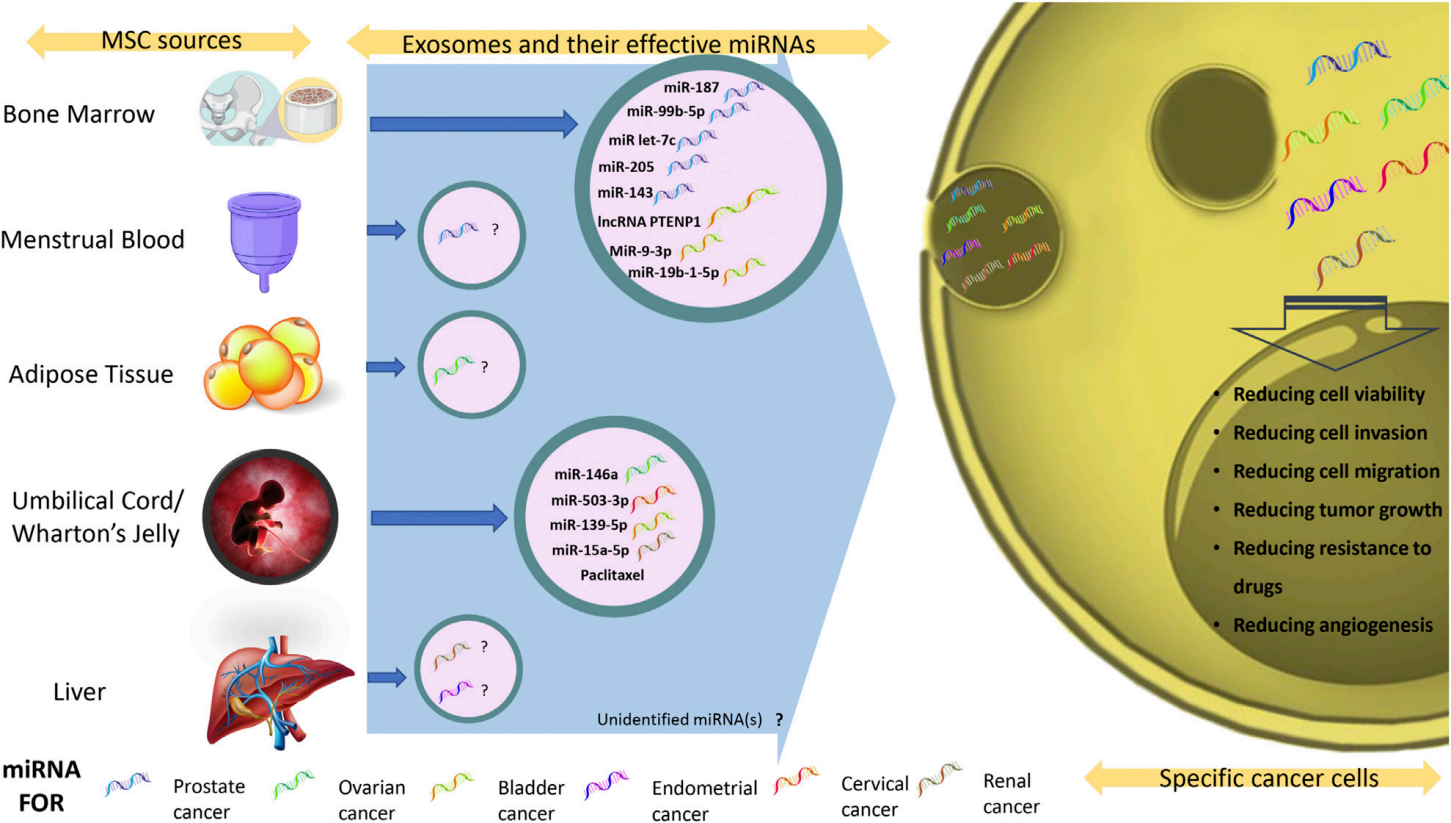
Exosomal miRNAs and their therapeutic effects against genitourinary neoplasms. Summary of all available exosomal miRNAs and their effect on genitourinary neoplasms.

The influence of exosomes produced by MSCs on genitourinary neoplasms, such as male, female, and urologic malignancies, has been compiled and presented in this review. The current review includes every study that has examined the use of MSC-derived exosomes in the treatment of urogenital neoplasms, which sums 16 studies. We have also shown how effectively exosomes can be used as potential new treatments in a separate table and figure as well (Table 1 and Figure 1). Extracellular vesicles, mesenchymal stem cells, and urogenital neoplasms were the search terms used in the PubMed data repository on November 2022. The inclusion of related studies was carried out without regarding chronological constraints and we omitted studies that have utilized total extracellular vesicles rather than exosomes specifically. It is worth mentioning the research’ increased publication frequency between 2016 and 2022 indicates that the topic is also receiving more attention.

**TABLE 1:** Mesenchymal stromal/stem cell- (MSC-) derived exosomes and their therapeutic mechanism on genitourinary neoplasms.

Urogenital cancer type	MSCs’ source	Effective RNAs/loaded drugs in exosomes	Direct or indirect Target(s) and effect(s)	Cancer cell line(s)	Therapeutic effect(s) mechanisms	References
Prostate Cancer	Bone marrow	miR-99b-5p	• IGF1R (Downregulation)	• LNCaP	• Reducing cell proliferation	Jiang et al.(2022)
				• DU145		
				• PC-3	• Reducing tumor growth (*in vivo*)	
	Bone marrow	miR let-7c	• Further data was not proposed by the study	• PC3	• Reducing cell proliferation	Kurniawati et al.(2022)
				• CWR22Rv1		
					• Reducing cell migration	
	Bone marrow	miR-187	• CD276 (JAK3-STAT3-Slug pathway) (Downregulation)	• 22Rv1	• Reducing cell viability	Li et al.(2022)
				• LNCaP		
				• Du145	• Reducing cell invasion	
				• PC3		
					• Reducing cell migration	
	Bone marrow	miR-205	• RHPN2 (Downregulation)	• LNCaP	• Reducing cell viability	Jiang et al.(2019)
					• Reducing cell invasion	
					• Reducing cell migration	
	Bone marrow	miR-143	• TFF3 (Downregulation)	• PC3	• Reducing cell viability	Che et al.(2019)
					• Reducing cell invasion	
					• Reducing cell migration	
	Menstrual blood	Further data was not proposed by the study	• NF-λB (Downregulation)	• PC3	• Reducing tumor hemoglobin content	Alcayaga-Miranda et al.(2016)
			• VEGF (Downregulation)		• Reducing the vascular density of tumor	
			• HIF-1β (Downregulation)			
Ovarian cancer	Human adipose tissue	Further data was not proposed by the study	• BAX (Upregulation)	• A2780	• Reducing cell viability	Reza et al.(2016)
			• CASP9 (Upregulation)	• SKOV-3		
			• CASP3 (Upregulation)		• Reducing proliferation	
			• BCL2 (Downregulation)		• Reducing colony formation	
	Human umbilical cord blood	miR-146a	• LAMC2 (Downregulation)	• SKOV3	• increasing the susceptibility of OCa cells to the chemotherapy	Qiu et al.(2020)
				• A2780		
			• PI3K/Akt (Downregulation)			
Cervical cancer	Wharton’s jelly	Paclitaxel (Drug)	• Bax (Upregulation)	• Hela	• Reducing cell viability	Abas et al.(2022)
			• BCL2 (Downregulation)		• Reducing resistance to chemotherapy	
			• clv-Cas-3 (Upregulation)			
			• clv-Cas-9 (Upregulation)			
			• TGF-β (Downregulation)			
			• catenin-β (Downregulation)			
Endometrial cancer	Human umbilical cord blood	miR-503-3p	• mesoderm-specific transcript (MEST) (Downregulation)	• HEC-1B	• Reducing cell growth	Pan et al.(2022)
				• RL95-2		
Bladder cancer	Human umbilical cord	miR-139-5p	• PRC1 (Downregulation)	• T24	• Reducing cell proliferation	Jia et al.(2021)
			• N-cadherin (Downregulation)		• Reducing cell invasion	
			• Vimentin (Downregulation)		• Reducing cell migration	
			• SNAIL (Downregulation)		• Reducing tumorigenesis *in vivo*	
			• Bcl-2 (Downregulation)			
			• PCNA (Downregulation)			
			• E-cadherin (Upregulation)			
			• Bax (Upregulation)			
	Bone marrow	lncRNA PTENP1	• miR-17	• 5637	• Reducing cell proliferation	Liu et al.(2022)
			• mir-17 (Sponging)	• T24		
			• SCARA5 (Upregulation)		• Reducing cell invasion	
					• Reducing cell migration	
					• Reducing tumorigenesis *in vivo*	
	Mouse bone marrow	MiR-9-3p	• ESM1	• BIU-87	• Reducing cell proliferation	Cai et al.(2019)
			• ESM1 (Downregulation)	• EJ		
				• T24	• Reducing cell invasion	
				• 5637		
				• UMUC-3		
					• Reducing cell migration	
					• Reducing tumorigenesis *in vivo*	
	Bone marrow	miR-19b-1-5p	• ABL2 (Downregulation)	• T24	• Reducing cell proliferation	Fu et al.(2021)
				• UC3		
			• Bcl-2 (Downregulation)	• 5637	• Reducing cell invasion	
				• J82		
			• MMP2 (Downregulation)		• Reducing cell migration	
			• MMP9 (Downregulation)			
Renal Cell cancer	Human liver	Further data was not proposed by the study	• miR-145	• Renal cells from carcinoma patients	• Reducing cell proliferation	Brossa et al.(2020)
		• miR-145	• miR-200		• Reducing cell invasion	
			• miR-200b (Upregulation)			
			• miR-200c (Upregulation)		• Reducing cell migration	
			• miR-Let7 (Upregulation)		• Reducing tumorigenesis *in vivo*	
			• miR-223 (Upregulation)			
			• EGFR (Downregulation)			
			• ZEB2 (Downregulation)			
			• MMP1 (Downregulation)			
Wilms tumor	Human umbilical cord	miR-15a-5p	• SEPT2 (Downregulation)	• G-401	• Reducing cell viability	Huang et al.(2022)
					• Reducing cell proliferation	
					• Reducing cell invasion	
					• Reducing cell migration	

## Male genitourinary neoplasms and MSCs derived exosomes

### Prostate cancer and MSCs derived exosomes

Secreted exosomes from MSCs are associated with their antitumor activity (Yeo et al., 2013). Messenger RNAs (mRNAs), microRNAs (miRNAs), and proteins may all be transported *via* exosomes (Lässer, 2012). MiRNAs are short, non-coding types of RNAs that often exhibit changes in gene expression in a variety of malignancies like prostate cancer (Sharma and Baruah, 2019).

In a study on patients with prostate cancer (PCa), it was revealed that expressions of hsa-let-7a-5p and hsa-miR-21-5p were elevated in the serum of the high-risk group after radiation therapy but hsa-miR-99b-5p was not substantially different (Malla et al., 2018). Further investigation revealed that miR-99b-5p was increased in exosomes produced from human bone marrow MSCs (hBM-MSCs) but downregulated in neoplastic prostate tissue. The underlying mechanism was proposed as by inhibiting IGF1R, hBM-MSCs-derived exosomes with miR-99b-5p overexpression may ameliorate the progression of PCa which might reveal higher therapeutic and prognostic potency of exosomes (Jiang et al., 2022).

Androgen deprivation therapy resistance and cancer recurrence are two characteristics of the progressive variant of malignant PCa namely Castration-resistant prostate cancer (CRPC) (Landstrom et al., 1994; Divrik et al., 2012). Recent research showed that miR-let-7c has tumor-suppressive properties by inhibiting the multiplication and expansion of CRPC-like cells as well. In CRPC-like cell lines (PC3 and CWR22Rv1), incubation with either intact MSC-EXOs or MSC exosomes with miR let-7c enrichment significantly decreased the proliferation and metastasis tendency of the cells (Kurniawati et al., 2022).

It was discovered that exosomal miR-187 from human bone marrow-derived MSCs (hBM-MSC) inhibited the malignant characteristics of PCa cells. It may be established that miR-187 inhibits the JAK3-STAT3-Slug pathway in PCa by targeting CD276. PCa cell aggressive behaviors were decreased by exosomes from hBM-MSC with overexpression of miR-187, suggesting that these exosomes may represent a feasible therapeutic target (Li et al., 2022).

The impact of exosomes produced from hBM-MSCs overexpressing miR-205 on the advancement of PCa was investigated, and the findings indicated that the transfer of miR-205 from hBMSCs to PCa cells by modified exosomes reduces multiplication, redistribution, and penetration of PCa cells through adhering to the 3′UTR of RHPN2 which led to its suppression. (Jiang et al., 2019).

According to research, TFF3 expression rises in PCa-related tissues and cells whereas miR-143 expression falls. This particular idea was utilized and it was demonstrated that exosomes from human BM-MSCs with overexpression of miR-143 may prevent PCa cells (PC3) from proliferating, migrating, or invading while promoting apoptosis by suppressing TFF3. This was further shown by the decrease in PCNA, MMP-2, and MMP-9 production levels in cells exposed to exosomal miR-143. Eventually, to elucidate underlying processes, more investigation utilizing *in vivo* models is suggested (Che et al., 2019).

Exosomes were shown to be responsible for extinguishing NF-κB activity and reducing reactive oxygen species (ROS) formation *in vitro*, according to research on cells. Also, The PC3 cells used in the angiogenesis plug experiment (*in vivo*) showed diminished angiogenic characteristics (loss of VEGF and HIF-1 tissue expression) and decreased hemoglobin content within the plugs, which is presumably repercussions of the lowered ROS generation generated by menstrual stem cells derived exosomes as well (Alcayaga-Miranda et al., 2016).

MiR-99b-5p, miR let-7c, miR-187, miR-205, miR-143, and MSC-EXOs have all been claimed to be capable of reducing PCa development. For PCa studies, researchers have preferred to use the PC3 and later the LNCaP cell lines. Also, bone marrow has been the primary source of extracting MSCs in PCa trials. A common route (JAK3-STAT3-Slug pathway), which is regarded to be helpful in reducing tumor development, may also get greater attention in future investigations.

## Female genitourinary neoplasms and MSCs derived exosomes

### Ovarian cancer and MSCs derived exosomes

Prior research suggested that MSCs are important in preventing the development of Ovarian cancer (OCa) (Khalil et al., 2019). Additionally, mature MSCs play a crucial role in cell signaling as important tumor-targeted conveyance agents by depositing EVs that are highly enriched with miRNAs (O'Brien et al., 2018). In terms of the most prevalent malignancies that result in mortality, OCa is in eighth place (Webb and Jordan, 2017; Wang et al., 2022).

Analysis indicated that overexpression excessive expression of the exosomal miRNA hsa-miR-124-3p resulted in the suppression of many cyclin-dependent kinases (CDKs), including CDK2, CDK4, and CDK6. Likewise, uptake of exosomes led to lower proliferation and colony formation in A2780 cells than exposure to human adipose MSC condition media (hA-MSC-CM) alone, proving that exosomes originating from hA-MSC-CM are significant contributors to the suppression of A2780 cell division. It was supposed that generated vesicles enhanced autophagy by upregulating a variety of pro-apoptotic signaling molecules, including BAX, CASP9, and CASP3, and downregulating the anti-apoptotic protein BCL2 (Reza et al., 2016).

A study found that hUC-MSCs could efficiently transfer miR-146a into released exosomes, increasing the susceptibility of OCa cells to the chemotherapeutic drugs docetaxel and taxane by lowering LAMC2 expression and blocking the PI3K/Akt signaling pathway (a characteristic for many cancer types). By examining the impact of miR-146a inhibitors on cancer cell growth and apoptosis, these findings were further confirmed with reversed consequences as well (Qiu et al., 2020).

The PI3K/Akt signaling pathway has been proven to be intriguing to target in future studies for minimizing resistance to commonly used treatments like chemotherapy, and other therapies may be evaluated in the same trend. As it is noticeable, two preferred cell lines for OCa research have been SKOV3 and A2780.

### Cervical neoplasm and MSCs derived exosomes

Exosomes from human umbilical cord Wharton’s jelly MSCs were used in a study to load Paclitaxel and test the exosomes’ effects on cervical cancer (Hela) cell lines. As a consequence, cancer cell death was accelerated by influencing the levels of Bax, BCL2, clv-Cas-3, and clv-Cas-9, and chemoresistance was decreased by affecting epithelial-mesenchymal transition (EMT)-related proteins (such as TGF-β and catenin-β) (Abas et al., 2022).

### Endometrial neoplasm and MSCs derived exosomes

In order to ascertain their role in EC cell biological processes, human endometrial cancer (EC) cell lines were co-cultured with Exosomes or treated with upregulated miR-503-3p or suppressed mesoderm-specific transcript (MEST) vectors. MiR-503-3p upregulation or MEST downregulation inhibited the biological activities of EC cells. Additionally, it was revealed that exosomes produced by human umbilical cord mesenchymal stem cell-derived exosomes (hUC-MSC) inhibited the development of EC cells, while exosomes with elevated miR-503-3p content from the same resource exhibited a more pronounced regulatory impact (Pan et al., 2022).

## Urologic neoplasms and MSCs derived exosomes

### Bladder neoplasm and MSCs derived exosomes

The gene PRC1 may be a novel target for miR-139-5p, according to recent research. Additionally, PRC1’s role as a carcinogen in bladder cancer cells has been confirmed. Results showed that deletion of PRC1 inhibited bladder cancer cells’ ability to proliferate and spread, as well as halted the induction of EMT, which was shown by changes in the production of E-cadherin, and Bax and decreases in the synthesis of N-cadherin, Vimentin, SNAIL, Bcl-2, and PCNA. hUC-MSC-derived exosomes’ effective delivery of miR-139-5p to T24 cells was verified as well. Additionally, the introduction of miR-139-5p from hUC-MSC-derived exosomes resulted in the reduction of bladder carcinogenesis in nude mice as well as the suppression of T24 cell growth, penetration, and expansion (Jia et al., 2021).

Exosomal long non-coding RNA (lncRNA) PTENP1, which is released by hBM-MSCs, inhibits the aggressive phenotypes of Bladder cancer cells (5637 and T24) by controlling the miR-17/SCARA5 axis. In order to promote the expression of SCARA5, exosomal lncRNA PTENP1 sponges miR-17 to prevent bladder cancer cell malignant behaviors as the mechanism (Liu et al., 2022).

Recently researchers used mouse BM-MSC-generated exosomes to suppress the survivability, metastasis, and invasion of bladder cancer cells while promoting apoptosis. MiR-9-3p was shown to be an efficient agent that targets ESM1 and has been shown to have anti-oncogenic effects on bladder neoplasia-specific cell lines (BIU-87, EJ, T24, 5637, and UMUC-3) and BALB/c nude mice model of bladder cancer. As the underlying mechanism, following the treatment of Exo-miR-nine to three, the protein expression levels of proliferation-associated proteins (Ki67 and PCNA) and invasion-associated indicators (MMP-2 and MMP-9) dropped significantly (Cai et al., 2019).

ABL2 is up-regulated while miR-19b-1-5p is down-regulated in bladder cancer tissues and cell lines, according to another research. Recently, the research revealed that BMSCs-derived exosomal miR-19b-1-5p could inhibit bladder cancer cell lines (T24, UC3, 5637, and J82) from migrating, proliferating, or invading while promoting cell death *via* inhibiting ABL2, Bcl-2, MMP2 and MMP9 (Fu et al., 2021).

The most favored cell lines for bladder tumor therapy with MSC-EXOs have been T24 and 5637. Cell proliferation, invasion, migration, and further *in vivo* experiments after using exosomes were the most applied confirming methods in these researches. While a study has revealed that targeting miRNAs may be successful instead of utilizing them to target other cellular markers, it is also considered a further step toward cell-free treatment. This specific strategy seems feasible in the future as the functions of exosomal miRNAs are becoming more understood.

### Renal cell neoplasm and MSCs derived exosomes

Stem cells with mesenchymal origin have shown promising results in renal cell neoplasm as well. In a study, researchers examined the impact of EVs produced from human liver stem cells (hL-SCs) with mesenchymal origin both *in vitro* and *in vivo*. In SCID mice, pretreatment of renal cancer stem cells with EVs prior to subcutaneous infusion slowed tumor initiation. By lowering tumor angiogenesis and triggering tumor cell death, hL-SCs-derived EVs dramatically reduced subcutaneous tumor development too. In tumor explants that had received treatment from EVs, upregulation of the miR-200 family members, miR-Let7, and miR-223 was obsereved, and the delivery of mir-145 by EVs was considered to be effective. Their target genes, such as EGFR, ZEB2, and MMP1, were inhibited with diminishing impacts on cell survival, invasion, and proliferation in transfected cancer stem cells eventually (Brossa et al., 2020).

### Wilms tumor and MSCs derived exosomes

Wilms tumor (WT), the most common kidney carcinoma in children, is an embryonic carcinoma without a definitive cure (Millar et al., 2017). According to a recent study, Septin 2 (SEPT2) loss-of-function in G-401 cells (WT cells) reduced proliferation, migration, and invasion while inducing apoptosis emphasizing the role of SEPT2 on tumorigenesis in WT. They proposed that SEPT2 could be targeted by miR-15a-5p-loaded hUC-MSCs derived exosomes that inhibited WT development significantly as a new means of therapeutics (Huang et al., 2022).

## Conclusion

Exosomes are single-membrane vesicles with a similar structure as cells and are generally considered the size between 30 and 200 nm (Chernyshev et al., 2015), a density of ∼1.1–1.2 g/ml (Raposo et al., 1996) and enrichment with marker proteins of CD81, CD63, and CD9 (Escola et al., 1998; Hemler, 2003). Exosomes also transport extracellular RNAs in active form to neighboring cells and tissues (Ratajczak et al., 2006). Small non-coding RNAs (ncRNAs), small nuclear RNAs (snRNAs), miRNAs, transfer RNAs (tRNAs), YRNAs, vault RNAs, repetitive element RNAs, and fragmented RNAs, including 3′mRNA remnants, appear to be abundant in exosomes (Shurtleff et al., 2017; Wei et al., 2017).

MSC-EXOs have recently been revealed to have the ability to repair tissue injuries and may be used to cure cancer (Keshtkar et al., 2018). Exosomes are smaller, less complex, and less immunogenic than their producing cells because they have fewer membrane-bound polypeptides (Lou et al., 2017). Thus, they do not cause tumor development or acute immunological rejection (Chen et al., 2011). Exosomes may be produced in significant quantities by MSCs *in vitro* (Yeo et al., 2013) and the overall safety of exosomes has been shown *in vivo* by several animal studies (Sun et al., 2016). This topic of stem cell therapy has received a great deal of interest, which may be attributed to all these aspects.

## Future insights

There are still unexplored areas that need to be studied in the future for even better outcomes. As (Jiang et al., 2022) stated, some already known downstream targets of miR-99b-5p like FGFR3 (Ding et al., 2021) and MFG-E8 (Lu et al., 2021) have the potential to be examined as potential therapeutics in the future (Kurniawati et al., 2022). also has shown the effectiveness of miR let-7c exosomes but the direct targets have not been detected by the study.

Other studies provided a distinctive insight as well. Although (Alcayaga-Miranda et al., 2016) did not provide a specific effective factor in exosomes, they utilized menstrual blood as a source of MSCs (Mens-SCs) which may possess superior qualities compared to other sources like bone marrow which seems to be preferred by researchers of this field by now. Menstrual blood stem cells secreted EVs may be more favorable for therapeutic use than whole cells either because of their ready-to-use potential, improved treatment efficacy, the introduction of fewer antigens, and prevention of immune responses. However, due to their fast clearance from the body, continuous treatment in substantial amounts is required (Bozorgmehr et al., 2020).

Furthermore, the difficulty of differentiating hBM-MSC is a limiting problem since it necessitates invasive surgery and donors’ ethical concerns, even though hBM-MSCs have received significant emphasis and dominating research (Chen et al., 2019). Mens-SCs can be delivered in huge volumes of cells by intravenous injection, they are safe and dependable (Wang et al., 2014), have shown no signs of tumor development or toxicity, and are capable of significantly slowing tumor development (Wang et al., 2017). Thus, they can be utilized more frequently in the future.

The majority of research has shown how miRNAs may improve tumorigenesis status, as demonstrated by Table 1. However, it has been hypothesized by (Liu et al., 2022) that miRNAs themselves may potentially be targeted. Similar therapeutic benefits may be obtained by employing lncRNAs, much as when miRNAs are first used for delivery. This concept is potentially beneficial and is one that might be adopted in the future.

In the end, it is noteworthy to highlight that different exosome cargos, including proteins, lipids, or RNAs, may contribute to the identified alleviating effects on cancer cells or tumors. Future research should be able to adequately demonstrate the effectiveness of a single exosome cargo if that is the focus of the investigation.

## Abbreviations

CDKs, cyclin-dependent kinases; PCa, Prostate cancer; CRPC, Castration-resistant prostate cancer; EC, Endometrial cancer; EVs, Extracellular vesicles; EXO, Exosome; hA-MSC-CM, human adipose tissue-derived mesenchymal stromal/stem cell condition media; hBM-MSC, Human bone marrow-derived mesenchymal stromal/stem cell; hL-SCs, Human liver stem cells; hUC-MSC, human umbilical cord-derived mesenchymal stromal/stem cell; lncRNA, Long non-coding RNA; Men-SCs, Menstrual blood-derived stem cells; MEST, mesoderm-specific transcript; miRNA, microRNA; mir, microRNA; MSC, Mesenchymal stromal/stem cell; MSC-EXO, Mesenchymal stromal/stem cell-derived exosome; OCa, Ovarian cancer; ROS, Reactive oxygen species; SEPT2, Septin 2; WT, Wilms tumor.
